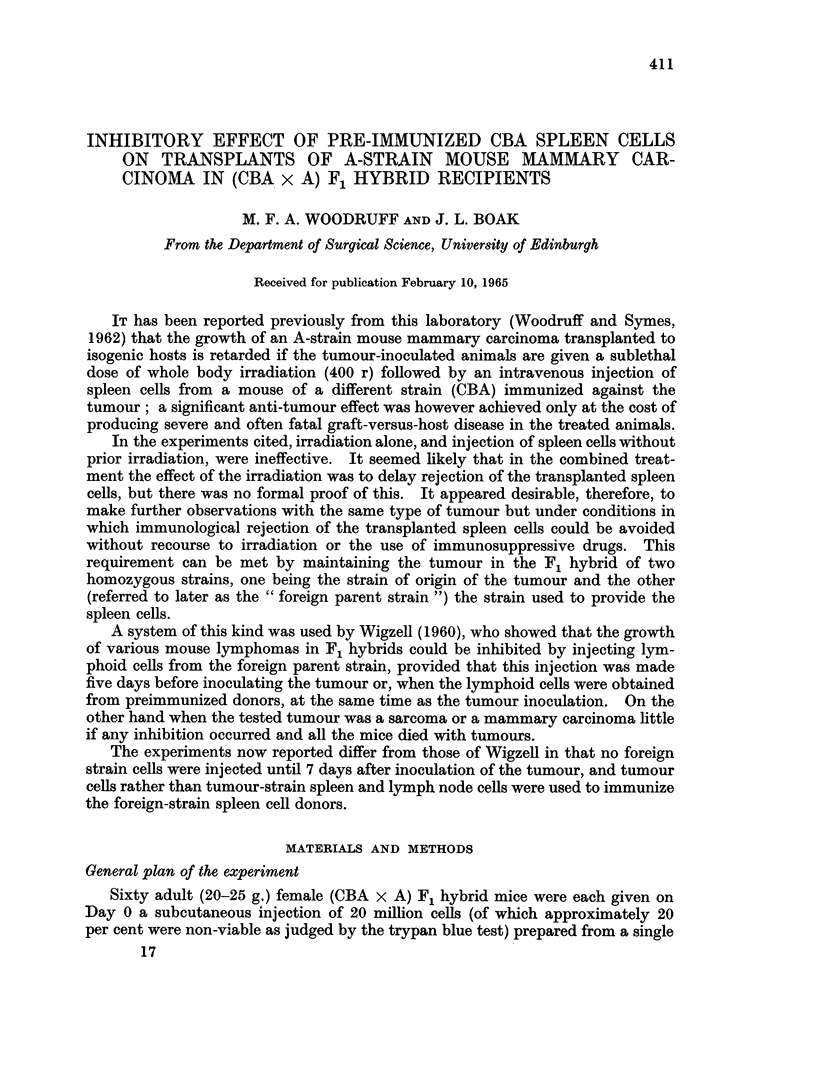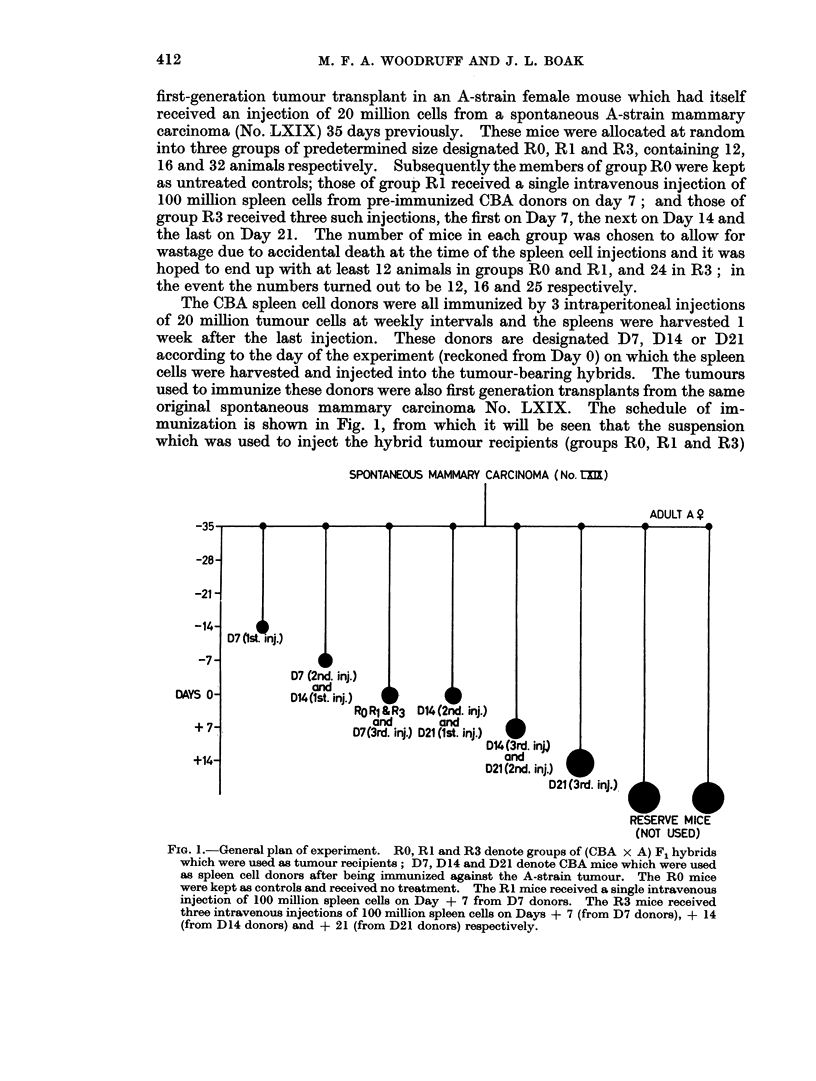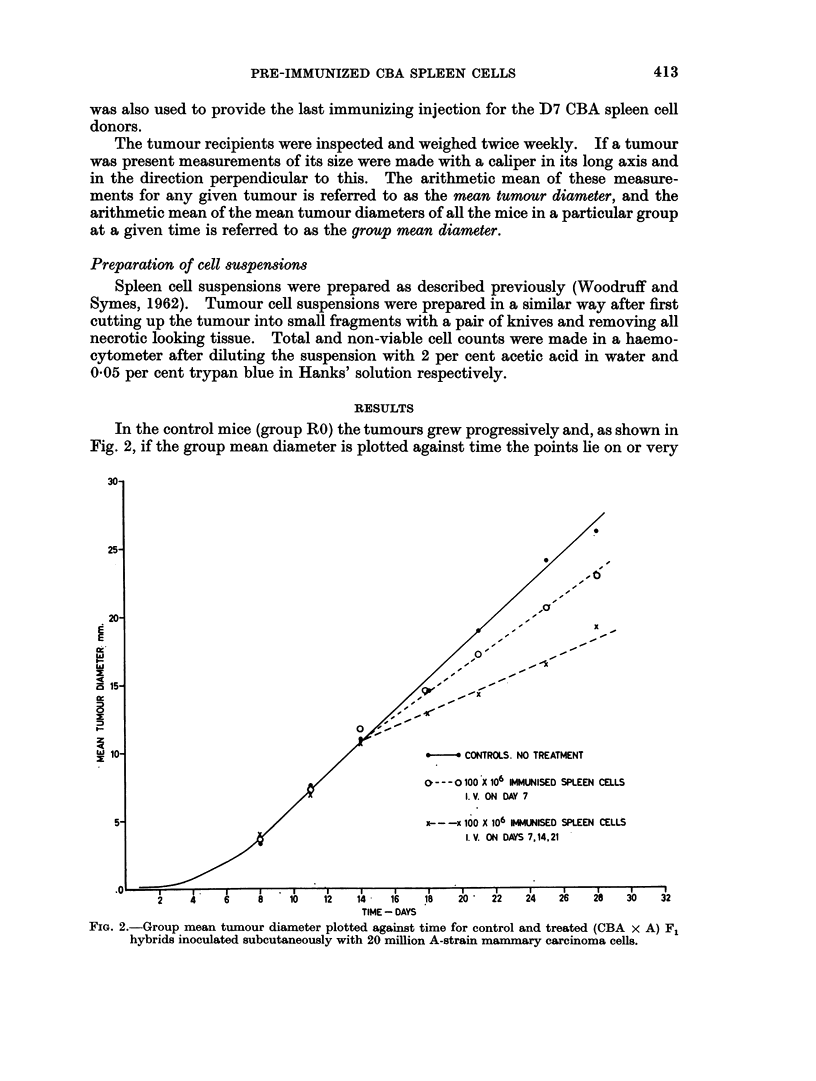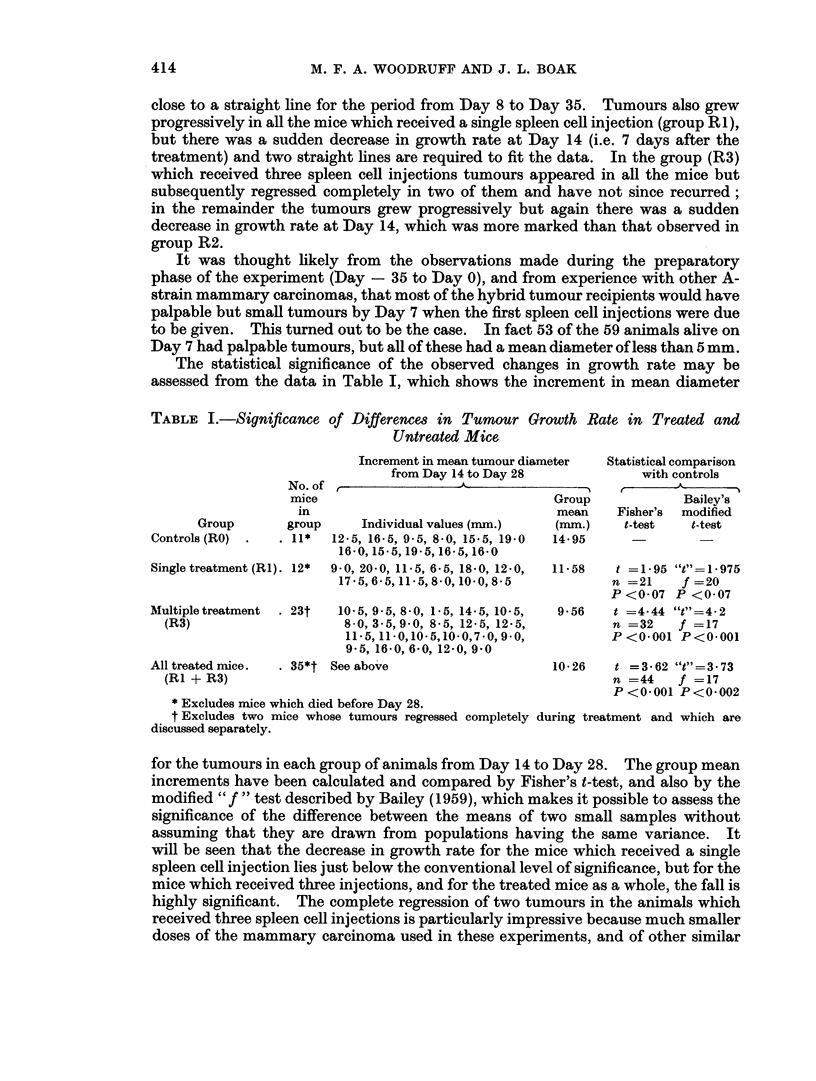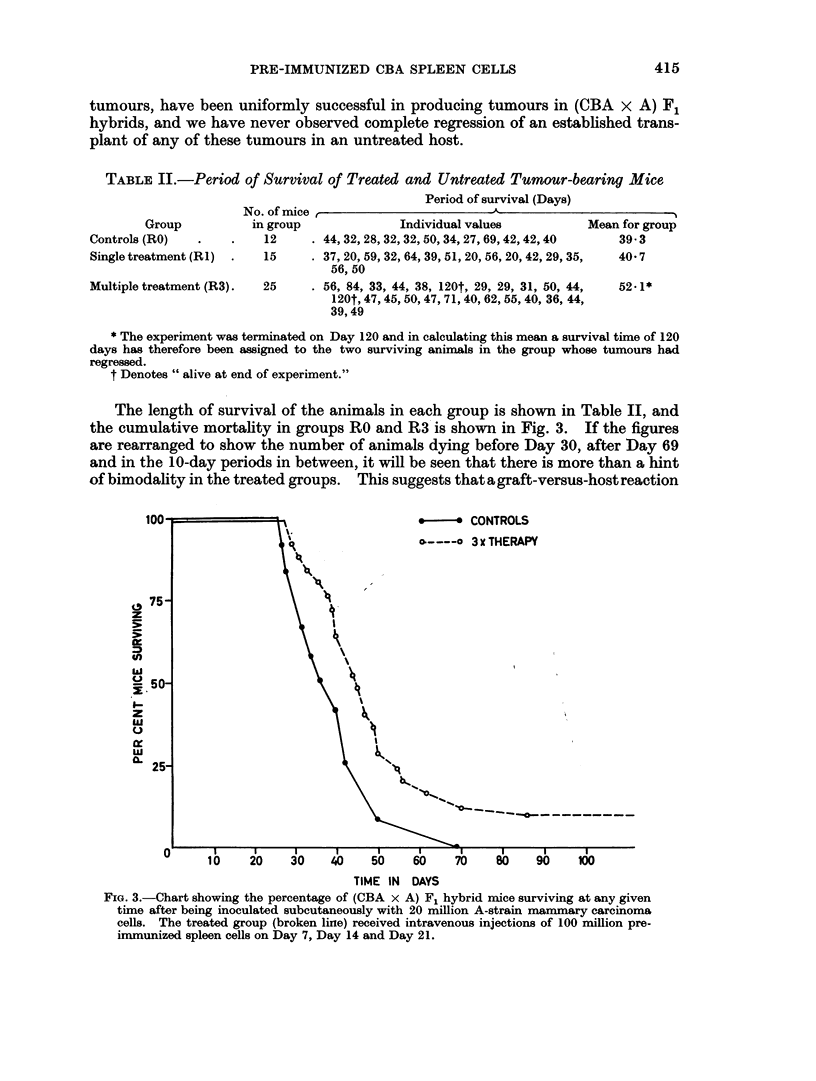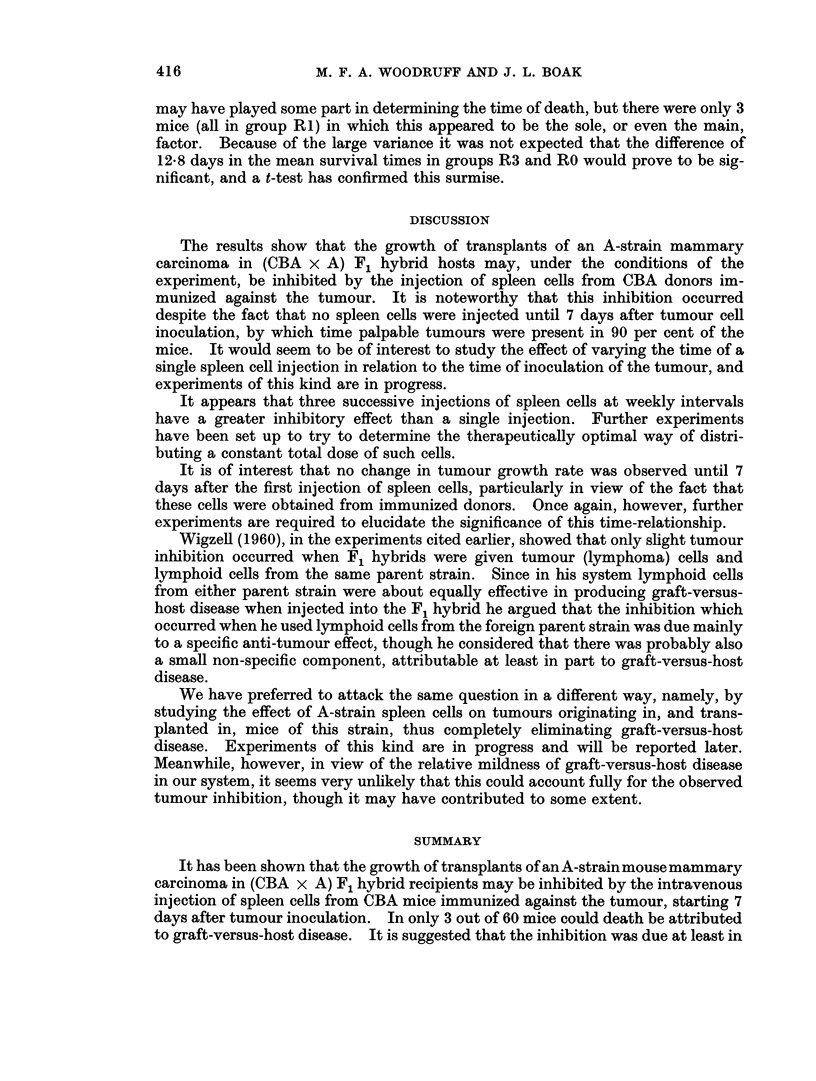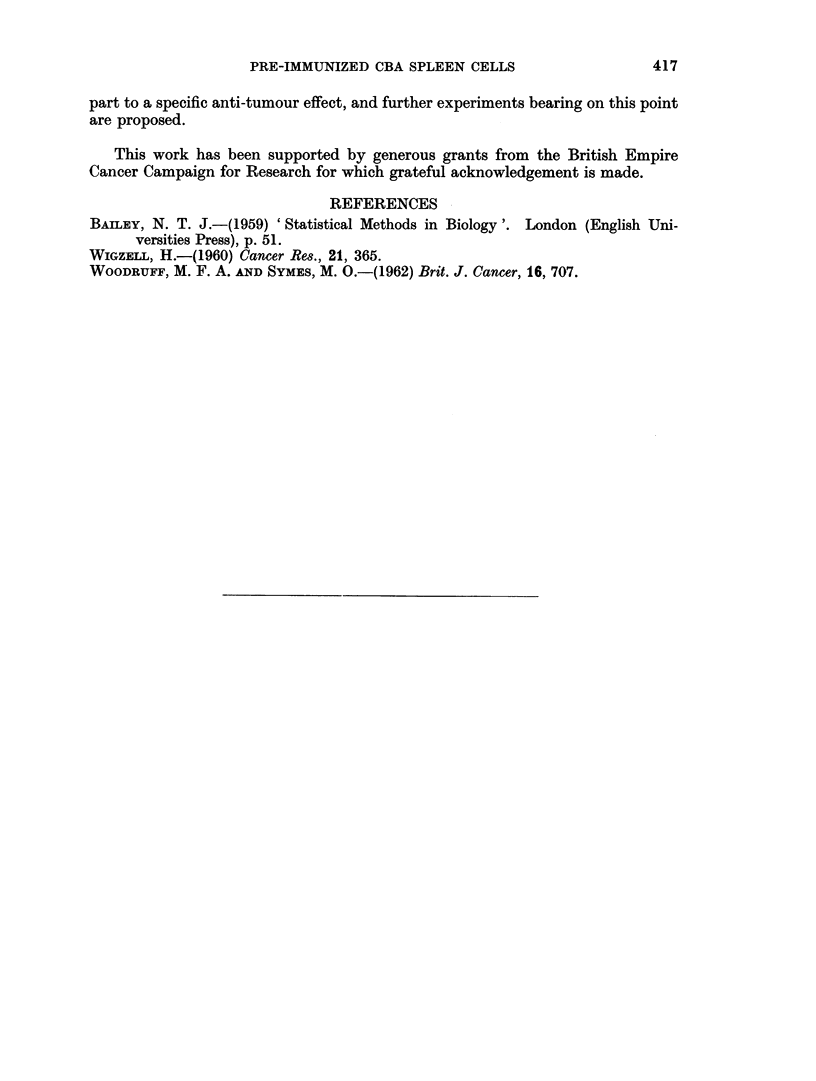# Inhibitory Effect of Pre-Immunized CBA Spleen Cells on Transplants of A-strain Mouse Mammary Carcinoma in (CBA × A) F_1_ Hybrid Recipients

**DOI:** 10.1038/bjc.1965.49

**Published:** 1965-06

**Authors:** M. F. A. Woodruff, J. L. Boak


### INHIBITORY EFFECT OF PRE-IMMUNIZED CBA SPLEEN CELLS ON TRANSPLANTS OF A-STRAIN MOUSE MAMMARY CARCINOMA IN (CBA × A) $F_1$ HYBRID RECIPIENTS

M. F. A. WOODRUFF AND J. L. BOAK

*From the Department of Surgical Science, University of Edinburgh*

Received for publication February 10, 1965

IT has been reported previously from this laboratory (Woodruff and Symes, 1962) that the growth of an A-strain mouse mammary carcinoma transplanted to isogenic hosts is retarded if the tumour-inoculated animals are given a sublethal dose of whole body irradiation (400 r) followed by an intravenous injection of spleen cells from a mouse of a different strain (CBA) immunized against the tumour ; a significant anti-tumour effect was however achieved only at the cost of producing severe and often fatal graft-versus-host disease in the treated animals.

In the experiments cited, irradiation alone, and injection of spleen cells without prior irradiation, were ineffective. It seemed likely that in the combined treatment the effect of the irradiation was to delay rejection of the transplanted spleen cells, but there was no formal proof of this. It appeared desirable, therefore, to make further observations with the same type of tumour but under conditions in which immunological rejection of the transplanted spleen cells could be avoided without recourse to irradiation or the use of immunosuppressive drugs. This requirement can be met by maintaining the tumour in the $F_1$ hybrid of two homozygous strains, one being the strain of origin of the tumour and the other (referred to later as the " foreign parent strain ") the strain used to provide the spleen cells.

A system of this kind was used by Wigzell (1960), who showed that the growth of various mouse lymphomas in $F_1$ hybrids could be inhibited by injecting lymphoid cells from the foreign parent strain, provided that this injection was made five days before inoculating the tumour or, when the lymphoid cells were obtained from preimmunized donors, at the same time as the tumour inoculation. On the other hand when the tested tumour was a sarcoma or a mammary carcinoma little if any inhibition occurred and all the mice died with tumours.

The experiments now reported differ from those of Wigzell in that no foreign strain cells were injected until 7 days after inoculation of the tumour, and tumour cells rather than tumour-strain spleen and lymph node cells were used to immunize the foreign-strain spleen cell donors.

### MATERIALS AND METHODS

### *General plan of the experiment*

Sixty adult (20–25 g.) female (CBA × A) $F_1$ hybrid mice were each given on Day 0 a subcutaneous injection of 20 million cells (of which approximately 20 per cent were non-viable as judged by the trypan blue test) prepared from a single first-generation tumour transplant in an A-strain female mouse which had itself received an injection of 20 million cells from a spontaneous A-strain mammary carcinoma (No. LXIX) 35 days previously. These mice were allocated at random into three groups of predetermined size designated R0, R1 and R3, containing 12, 16 and 32 animals respectively. Subsequently the members of group R0 were kept as untreated controls; those of group R1 received a single intravenous injection of 100 million spleen cells from pre-immunized CBA donors on day 7 ; and those of group R3 received three such injections, the first on Day 7, the next on Day 14 and the last on Day 21. The number of mice in each group was chosen to allow for wastage due to accidental death at the time of the spleen cell injections and it was hoped to end up with at least 12 animals in groups R0 and R1, and 24 in R3 ; in the event the numbers turned out to be 12, 16 and 25 respectively.

The CBA spleen cell donors were all immunized by 3 intraperitoneal injections of 20 million tumour cells at weekly intervals and the spleens were harvested 1 week after the last injection. These donors are designated D7, D14 or D21 according to the day of the experiment (reckoned from Day 0) on which the spleen cells were harvested and injected into the tumour-bearing hybrids. The tumours used to immunize these donors were also first generation transplants from the same original spontaneous mammary carcinoma No. LXIX. The schedule of immunization is shown in Fig. 1, from which it will be seen that the suspension which was used to inject the hybrid tumour recipients (groups R0, R1 and R3)

FIG. 1.—General plan of experiment. R0, R1 and R3 denote groups of (CBA × A) $F_1$ hybrids which were used as tumour recipients ; D7, D14 and D21 denote CBA mice which were used as spleen cell donors after being immunized against the A-strain tumour. The R0 mice were kept as controls and received no treatment. The R1 mice received a single intravenous injection of 100 million spleen cells on Day + 7 from D7 donors. The R3 mice received three intravenous injections of 100 million spleen cells on Days + 7 (from D7 donors), + 14 (from D14 donors) and + 21 (from D21 donors) respectively.

was also used to provide the last immunizing injection for the D7 CBA spleen cell donors.

The tumour recipients were inspected and weighed twice weekly. If a tumour was present measurements of its size were made with a caliper in its long axis and in the direction perpendicular to this. The arithmetic mean of these measurements for any given tumour is referred to as the *mean tumour diameter*, and the arithmetic mean of the mean tumour diameters of all the mice in a particular group at a given time is referred to as the *group mean diameter*.

*Preparation of cell suspensions*

Spleen cell suspensions were prepared as described previously (Woodruff and Symes, 1962). Tumour cell suspensions were prepared in a similar way after first cutting up the tumour into small fragments with a pair of knives and removing all necrotic looking tissue. Total and non-viable cell counts were made in a haemocytometer after diluting the suspension with 2 per cent acetic acid in water and 0·05 per cent trypan blue in Hanks' solution respectively.

### RESULTS

In the control mice (group R0) the tumours grew progressively and, as shown in Fig. 2, if the group mean diameter is plotted against time the points lie on or very

FIG. 2.—Group mean tumour diameter plotted against time for control and treated (CBA × A) $F_1$ hybrids inoculated subcutaneously with 20 million A-strain mammary carcinoma cells.

close to a straight line for the period from Day 8 to Day 35. Tumours also grew progressively in all the mice which received a single spleen cell injection (group R1), but there was a sudden decrease in growth rate at Day 14 (i.e. 7 days after the treatment) and two straight lines are required to fit the data. In the group (R3) which received three spleen cell injections tumours appeared in all the mice but subsequently regressed completely in two of them and have not since recurred; in the remainder the tumours grew progressively but again there was a sudden decrease in growth rate at Day 14, which was more marked than that observed in group R2.

It was thought likely from the observations made during the preparatory phase of the experiment (Day − 35 to Day 0), and from experience with other A-strain mammary carcinomas, that most of the hybrid tumour recipients would have palpable but small tumours by Day 7 when the first spleen cell injections were due to be given. This turned out to be the case. In fact 53 of the 59 animals alive on Day 7 had palpable tumours, but all of these had a mean diameter of less than 5 mm.

The statistical significance of the observed changes in growth rate may be assessed from the data in Table I, which shows the increment in mean diameter

TABLE I.—*Significance of Differences in Tumour Growth Rate in Treated and Untreated Mice*

| Group | No. of mice in group | Increment in mean tumour diameter from Day 14 to Day 28: Individual values (mm.) | Increment in mean tumour diameter from Day 14 to Day 28: Group mean (mm.) | Statistical comparison with controls: Fisher's $t$-test | Statistical comparison with controls: Bailey's modified $t$-test |
|---|---|---|---|---|---|
| Controls (R0) | 11* | 12·5, 16·5, 9·5, 8·0, 15·5, 19·0 16·0, 15·5, 19·5, 16·5, 16·0 | 14·95 | — | — |
| Single treatment (R1) | 12* | 9·0, 20·0, 11·5, 6·5, 18·0, 12·0, 17·5, 6·5, 11·5, 8·0, 10·0, 8·5 | 11·58 | $t$ = 1·95<br>$n$ = 21<br>$P < 0·07$ | "$t$" = 1·975<br>$f$ = 20<br>$P < 0·07$ |
| Multiple treatment (R3) | 23† | 10·5, 9·5, 8·0, 1·5, 14·5, 10·5, 8·0, 3·5, 9·0, 8·5, 12·5, 12·5, 11·5, 11·0, 10·5, 10·0, 7·0, 9·0, 9·5, 16·0, 6·0, 12·0, 9·0 | 9·56 | $t$ = 4·44<br>$n$ = 32<br>$P < 0·001$ | "$t$" = 4·2<br>$f$ = 17<br>$P < 0·001$ |
| All treated mice (R1 + R3) | 35*† | See above | 10·26 | $t$ = 3·62<br>$n$ = 44<br>$P < 0·001$ | "$t$" = 3·73<br>$f$ = 17<br>$P < 0·002$ |

* Excludes mice which died before Day 28.

† Excludes two mice whose tumours regressed completely during treatment and which are discussed separately.

for the tumours in each group of animals from Day 14 to Day 28. The group mean increments have been calculated and compared by Fisher's $t$-test, and also by the modified "$f$" test described by Bailey (1959), which makes it possible to assess the significance of the difference between the means of two small samples without assuming that they are drawn from populations having the same variance. It will be seen that the decrease in growth rate for the mice which received a single spleen cell injection lies just below the conventional level of significance, but for the mice which received three injections, and for the treated mice as a whole, the fall is highly significant. The complete regression of two tumours in the animals which received three spleen cell injections is particularly impressive because much smaller doses of the mammary carcinoma used in these experiments, and of other similar tumours, have been uniformly successful in producing tumours in (CBA × A) $F_1$ hybrids, and we have never observed complete regression of an established transplant of any of these tumours in an untreated host.

TABLE II.—*Period of Survival of Treated and Untreated Tumour-bearing Mice*

| Group | No. of mice in group | Period of survival (Days): Individual values | Mean for group |
|---|---|---|---|
| Controls (R0) | 12 | 44, 32, 28, 32, 32, 50, 34, 27, 69, 42, 42, 40 | 39·3 |
| Single treatment (R1) | 15 | 37, 20, 59, 32, 64, 39, 51, 20, 56, 20, 42, 29, 35, 56, 50 | 40·7 |
| Multiple treatment (R3) | 25 | 56, 84, 33, 44, 38, 120†, 29, 29, 31, 50, 44, 120†, 47, 45, 50, 47, 71, 40, 62, 55, 40, 36, 44, 39, 49 | 52·1* |

* The experiment was terminated on Day 120 and in calculating this mean a survival time of 120 days has therefore been assigned to the two surviving animals in the group whose tumours had regressed.

† Denotes " alive at end of experiment."

The length of survival of the animals in each group is shown in Table II, and the cumulative mortality in groups R0 and R3 is shown in Fig. 3. If the figures are rearranged to show the number of animals dying before Day 30, after Day 69 and in the 10-day periods in between, it will be seen that there is more than a hint of bimodality in the treated groups. This suggests that a graft-versus-host reaction

FIG. 3.—Chart showing the percentage of (CBA × A) $F_1$ hybrid mice surviving at any given time after being inoculated subcutaneously with 20 million A-strain mammary carcinoma cells. The treated group (broken line) received intravenous injections of 100 million pre-immunized spleen cells on Day 7, Day 14 and Day 21.

may have played some part in determining the time of death, but there were only 3 mice (all in group R1) in which this appeared to be the sole, or even the main, factor. Because of the large variance it was not expected that the difference of 12·8 days in the mean survival times in groups R3 and R0 would prove to be significant, and a *t*-test has confirmed this surmise.

### DISCUSSION

The results show that the growth of transplants of an A-strain mammary carcinoma in (CBA × A) $F_1$ hybrid hosts may, under the conditions of the experiment, be inhibited by the injection of spleen cells from CBA donors immunized against the tumour. It is noteworthy that this inhibition occurred despite the fact that no spleen cells were injected until 7 days after tumour cell inoculation, by which time palpable tumours were present in 90 per cent of the mice. It would seem to be of interest to study the effect of varying the time of a single spleen cell injection in relation to the time of inoculation of the tumour, and experiments of this kind are in progress.

It appears that three successive injections of spleen cells at weekly intervals have a greater inhibitory effect than a single injection. Further experiments have been set up to try to determine the therapeutically optimal way of distributing a constant total dose of such cells.

It is of interest that no change in tumour growth rate was observed until 7 days after the first injection of spleen cells, particularly in view of the fact that these cells were obtained from immunized donors. Once again, however, further experiments are required to elucidate the significance of this time-relationship.

Wigzell (1960), in the experiments cited earlier, showed that only slight tumour inhibition occurred when $F_1$ hybrids were given tumour (lymphoma) cells and lymphoid cells from the same parent strain. Since in his system lymphoid cells from either parent strain were about equally effective in producing graft-versus-host disease when injected into the $F_1$ hybrid he argued that the inhibition which occurred when he used lymphoid cells from the foreign parent strain was due mainly to a specific anti-tumour effect, though he considered that there was probably also a small non-specific component, attributable at least in part to graft-versus-host disease.

We have preferred to attack the same question in a different way, namely, by studying the effect of A-strain spleen cells on tumours originating in, and transplanted in, mice of this strain, thus completely eliminating graft-versus-host disease. Experiments of this kind are in progress and will be reported later. Meanwhile, however, in view of the relative mildness of graft-versus-host disease in our system, it seems very unlikely that this could account fully for the observed tumour inhibition, though it may have contributed to some extent.

### SUMMARY

It has been shown that the growth of transplants of an A-strain mouse mammary carcinoma in (CBA × A) $F_1$ hybrid recipients may be inhibited by the intravenous injection of spleen cells from CBA mice immunized against the tumour, starting 7 days after tumour inoculation. In only 3 out of 60 mice could death be attributed to graft-versus-host disease. It is suggested that the inhibition was due at least in part to a specific anti-tumour effect, and further experiments bearing on this point are proposed.

This work has been supported by generous grants from the British Empire Cancer Campaign for Research for which grateful acknowledgement is made.

### REFERENCES

BAILEY, N. T. J.—(1959) 'Statistical Methods in Biology'. London (English Universities Press), p. 51.
WIGZELL, H.—(1960) *Cancer Res.*, **21**, 365.
WOODRUFF, M. F. A. AND SYMES, M. O.—(1962) *Brit. J. Cancer*, **16**, 707.